# The Relationship between Embryonic Development and the Efficiency of Target Mutations in Porcine Endogenous Retroviruses (PERVs) *Pol* Genes in Porcine Embryos

**DOI:** 10.3390/ani9090593

**Published:** 2019-08-22

**Authors:** Maki Hirata, Manita Wittayarat, Takayuki Hirano, Nhien Thi Nguyen, Quynh Anh Le, Zhao Namula, Mokhamad Fahrudin, Fuminori Tanihara, Takeshige Otoi

**Affiliations:** 1 Faculty of Bioscience and Bioindustry, Tokushima University, Myozai-gun, Tokushima 7793233, Japan; 2 Faculty of Veterinary Science, Prince of Songkla University, Hat Yai, Songkhla 90110, Thailand; 3 Faculty of Veterinary Science, Guangdong Ocean University, Zhanjiang, Guangdong 524005, China; 4 Faculty of Veterinary Science, Bogor Agricultural University, Dramaga, Bogor 16680, Indonesia

**Keywords:** electroporation, GEEP, genome editing, PERV, *pol* gene

## Abstract

**Simple Summary:**

Pigs with porcine endogenous retrovirus (PERV) inactivation are preferable donor sources for xenotransplantation because the PERV may act as an infectious pathogen for humans who receive pig organ xenotransplantation. However, inactivation of the PERV *pol* gene in porcine cells reportedly affects cell growth. The present study clarified the relationship between the mutation of the PERV *pol* gene in porcine embryos and their development. Three different gRNAs targeting the PERV *pol* gene were introduced into porcine zygotes by genome editing using electroporation of the Cas9 protein (GEEP) system. The results demonstrated a negative relationship between the embryonic development and the efficiency of target mutations in the PERV *pol* gene of the porcine embryos.

**Abstract:**

Porcine endogenous retrovirus (PERV) is a provirus found in the pig genome that may act as an infectious pathogen in humans who receive pig organ xenotransplantation. Inactivation of the PERV *pol* gene in porcine cells reportedly affects cell growth. Therefore, the mutation of PERV *pol* gene in porcine embryos using genome editing may affect the embryonic development. The present study was carried out to investigate the relationship between the mutation of the PERV *pol* gene in porcine embryos and their development. We introduced, either alone or in combination, three different gRNAs (gRNA1, 2, and 3) into porcine zygotes by genome editing using electroporation of the Cas9 protein (GEEP) system. All three gRNAs targeted the PERV *pol* gene, and we assessed their effects on porcine embryonic development. Our results showed that the blastocyst formation rates of zygotes electroporated with gRNA3—alone and in combination—were significantly lower (*p* < 0.05) than those of zygotes electroporated with gRNA1. The mutation rates assessed by the PERV *pol* gene target site sequencing in individual blastocysts and pooled embryos at the 2-to-8-cell stage did not differ among the three gRNAs. However, the frequency of indel mutations in mutant embryos at the 2-to-8-cell stage trended higher in the embryos electroporated with gRNA3 alone and in combination. Embryonic development may be affected by gRNAs that induce high-frequency indel mutations.

## 1. Introduction

Organ transplantation is an established, effective, and safe tool for treating patients with end-stage organ failure. Xenotransplantation (cross-species transplantation) has evolved as an attempt to resolve critical shortages of human organs [1]. Genetic engineering and cloning technologies allow porcine tissues that are used for xenotransplantation to be protected from human immune responses [1]. However, the health and safety of xenotransplantation is an area of ongoing concern. In particular, porcine tissues may cause zoonosis in the recipient following xenotransplantation [2,3].

Endogenous retroviruses are viral elements integrated and transmitted vertically through the germline, and are then transmitted to subsequent generations [4,5]. Porcine endogenous retrovirus (PERV) may act as an infectious disease in humans that should be closely monitored during and after xenotransplantation. PERVs have single-stranded RNA (ssRNA) genomes, which contain *gag*, *pol*, and *env* genes [6]. The *gag* gene encodes a structural protein, and the *env* gene encodes an envelope protein that plays an important role in the infection of host cells [7,8,9]. The *pol* gene encodes protease, reverse transcriptase, and integrase, and plays a crucial role in the replication cycle of retroviruses because it transcribes genomic RNA into double-stranded DNA (dsDNA), called the provirus, which is subsequently integrated into the genome of the host cell [2,3,4,6,10]. The PERV copy numbers vary among different breeds of pig, and among multiple organs within each animal [3]. European wild boar and Chinese miniature pigs contain fewer PERV copies than Western breeds [11,12]. Pigs with low PERV copy number or PERV inactivation are preferable donor sources for xenotransplantation.

Clustered regularly interspaced short palindromic repeats-associated protein 9 nuclease (CRISPR/Cas9) is a powerful genome-editing tool used with animals and plants [13,14]. Recently, Yang et al. [15] reported that all PERVs in a porcine kidney epithelial cell line could be inactivated using the CRISPR/Cas9 system. However, DNA cleavage via the CRISPR/Cas9 system at multiple PERV sites in the genome of porcine cells may trigger DNA-damage-induced senescence or apoptosis, resulting in poor growth of the modified cells [16]. In a previous study, we demonstrated that the type of gRNA affected the development of porcine embryos edited by the CRISPR/Cas9 system. This indicated that the targeting of the genomic region by gRNA caused embryonic lethality [17]. However, there is no information on the development and target mutations in the PERV *pol* gene of porcine embryos after the introduction of the CRISPR/Cas9 system by electroporation. 

To clarify the effects of PERV *pol* gene mutation on the development of porcine embryos, we investigated the relationship between the embryonic development and the efficiency of target mutations in the PERV *pol* gene on porcine zygotes edited by three different gRNAs, either alone or in combination, using the genome editing by electroporation of the Cas9 protein (GEEP) system.

## 2. Materials and Methods

### 2.1. General

All procedures were approved by the Animal Research Committee of Tokushima University (ethical code: T28-21).

### 2.2. Oocyte Collection, in Vitro Maturation, Fertilization, and Embryo Culture

Oocyte collection, in vitro maturation, fertilization, and embryo culture were carried out as described previously [13]. We obtained about 200 ovaries from prepubertal crossbred gilts (Landrace × Large White × Duroc breeds) at a local slaughterhouse. Ovaries were washed three times with modified phosphate-buffered saline (m-PBS; Nihonzenyaku, Fukushima, Japan) supplemented with 100 IU/mL penicillin G potassium (Meiji, Tokyo, Japan) and 0.1 mg/mL streptomycin sulfate (Meiji). The cumulus-oocyte complexes (COCs) were collected from 3–6-mm follicles using a surgical blade. The COCs with a uniform ooplasm and compact cumulus cell mass were cultured in maturation medium at 39 °C in a humidified incubator containing 5% CO_2_. The maturation medium consisted of 25 mM HEPES tissue culture medium 199 with Earle’s salts (TCM 199; Invitrogen Co., Carlsbad, CA, USA), supplemented with 10% (*v*/*v*) porcine follicular fluid, 0.6 mM cysteine (Sigma-Aldrich, St. Louis, MO, USA), 50 µM sodium pyruvate (Sigma-Aldrich), 2 mg/mL d-sorbitol (Wako Pure Chemical Industries Ltd., Osaka, Japan), 50 μM β-mercaptoethanol (Wako Pure Chemical Industries Ltd.), 10 IU/mL equine chorionic gonadotropin (Kyoritu Seiyaku, Tokyo, Japan), 10 IU/mL human chorionic gonadotropin (Kyoritu Seiyaku, Tokyo, Japan), and 50 µg/mL gentamicin (Sigma-Aldrich, St. Louis, MO, USA). After maturation for 20 to 22 h, the COCs were cultured for an additional 24 h in maturation medium without hormones under the same conditions.

For in vitro fertilization (IVF), frozen–thawed boar spermatozoa (Landrace breed) were transferred into 6 mL of porcine fertilization medium (PFM; Research Institute for the Functional Peptides Co., Yamagata, Japan) and washed by centrifuging at 500× *g* for 5 min. The pelleted spermatozoa were resuspended in PFM and adjusted to 5 × 10^6^ cells/mL. Then, the matured oocytes were transferred to the sperm-containing PFM and co-incubated in a humidified incubator containing 5% CO_2_, 5% O_2_, and 90% N_2_ for 5 h at 39 °C. 

After IVF, the zygotes were washed with pig zygote medium (PZM-5; Research Institute for the Functional Peptides Co.) and were cultured continuously in vitro at 39 °C in a humidified incubator containing 5% CO_2_, 5% O_2_, and 90% N_2_. All of the cleaved embryos were transferred into 100-µL droplets of porcine blastocyst medium (PBM, Research Institute for the Functional Peptides Co.) 72 h after insemination. The embryos were subsequently cultured for an additional 4 days to evaluate their ability to develop to the blastocyst stage. 

### 2.3. Electroporation

**Figure 1:**
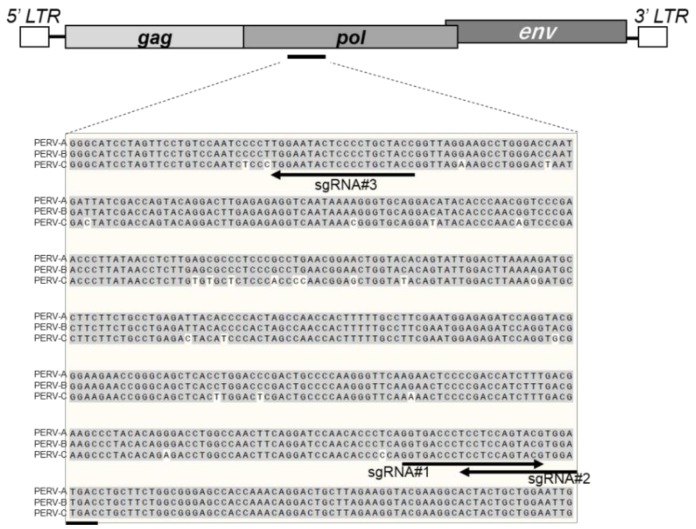
Genomic structure of porcine endogenous retrovirus (PERV) and gRNA locations targeting the *pol* gene. Three types of gRNAs (arrows) are shown in the alignments of PERV-A, PERV-B, and PERV-C *pol* genes.

**Figure 2:**
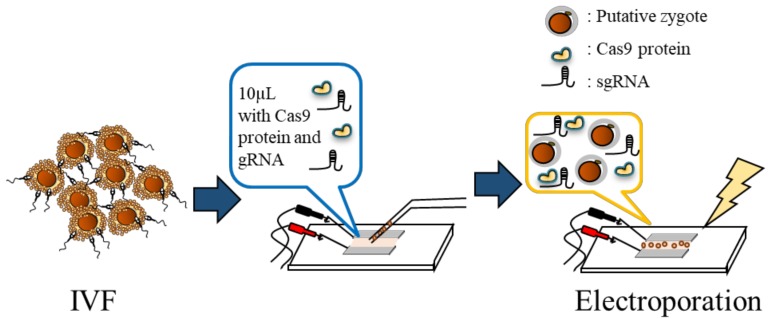
Schemes of genome editing using the electroporation method. Approximately 30–40 putative zygotes were placed in a line in the electrode gap, in a chamber slide filled with 10 μL of Opti-MEM I solution with Cas9 protein and gRNA, and were then electroporated by 25 V/mm, 1 ms, and five repeats. IVF: in vitro fertilization.

Three different gRNAs targeting the PERV *pol* gene were designed; the target sequences were as follows: (1) sgRNA1: 5′- GGTGACCCTCCTCCAGTACG-3′, (2) sgRNA2: 5′- GTCATCCACGTACTGGAGGA-3′, and (3) sgRNA3: 5′- GGTAGCAGGGGAGTATTCCA-3′. gRNAs were designed using the CRISPR direct webtool (https://crispr.dbcls.jp/) [18]. The PAM sequence was set as NGG. We selected the gRNAs which targeted the sequence conserved in PERV-A, PERV-B, and PERV-C (Figure 1), because PERV-A and PERV-B have been reported to infect several mammalian species, including human [10,19]. Moreover, PERV-A/C resulting from the recombination of subtypes A and C has been demonstrated to be infectious to human cells and to show higher replication titers than PERV-A [20]. Since the Cas9 protein has been reported as intolerable for mismatches at the 3’ end of the gRNAs [21,22], we confirmed that 12 bases at the 3’ end of gRNA had no identical sequence in the pig genome other than the region expected for the PERV *pol* gene to minimize the possibility of the off-target effect using the COSMID webtool (https://crispr.bme.gatech.edu/) [23]. Electroporation was performed 13 h after the initiation of IVF as described previously [24]. Briefly, an electrode (LF501PT1-20; BEX, Tokyo, Japan) was connected to a CUY21EDIT II electroporator (BEX, Tokyo, Japan) and placed under a stereoscopic microscope. The putative zygotes (approximately 30–40 zygotes) were washed with Opti-MEM I solution (Thermo Fisher Scientific K.K., Tokyo, Japan) and placed in a line in the electrode gap, in a chamber slide filled with 10 μL of Opti-MEM I solution with 100 ng/µL of Cas9 protein and 10 ng/µL of gRNA targeting the PERV *pol* gene. They were then electroporated by 25 V/mm, 1 ms, and five repeats (Figure 2). After electroporation, the zygotes were cultured to examine the effect of different gRNAs targeting the PERV *pol* gene on the development of electroporated zygotes. We demonstrated that there was no difference in the embryonic survival after in vitro culture between electroporation procedures with and without gRNA and Cas9 protein [25]. Thus, as a control, some zygotes were cultured with PZM-5 and PBM for 7 days without performing electroporation.

### 2.4. Analysis of Targeted Gene Sequence

We analyzed the frequencies of base insertions or deletions (indels) in the PERV *pol* gene of individual blastocysts and pooled embryos at the 2-to-8-cell stage in order to compare the efficiency of target mutations in the embryos among the three gRNAs groups. The embryos at the 2-to-8-cell stage in each gRNA group were randomly collected 3 days after the electroporation treatment. The total number of blastomeres in the collected embryos per sample was adjusted to 16 in order to avoid cleavage stage bias. The genomic DNA of embryos was extracted using a heat treatment in 50 mM NaOH after neutralization. The DNA samples were subjected to PCR using Quick Taq HS DyeMix (Toyobo, Osaka, Japan) according to the manufacturer’s instructions. Primers used for amplification were: sgRNA1, 2: 5′- GATGCCTTCTTCTGCCTGAG -3′ (forward) and 5′ TTGGTTAGCGGGTAGAGTGG -3′ (reverse); sgRNA3: 5′- TTGAATAACCTGTGGGGGAA -3′ (forward) and 5′- TACTGGAGGAGGGTCACCTG -3′ (reverse). After purification of PCR products with a Fast Gene Gel/PCR Extraction Kit (Nippon Genetics, Tokyo, Japan), we analyzed the target region sequences using Sanger sequencing with a BigDye Terminator Cycle Sequencing Kit version 3.1 (Thermo Fisher Scientific K.K., Tokyo, Japan) in an ABI 3500 genetic analyzer (Thermo Fisher Scientific K.K., Tokyo, Japan). We used a Tracking of Indels by Decomposition (TIDE; https://tide.deskgen.com/) bioinformatics package to quantify the frequency of indel mutation events in embryos electroporated with PERV *pol* gRNA [26]. According to the target region sequences, they were classified as “wild-type” or “mosaic mutant,” in which the presence of different alleles with wild-type alleles was considered mosaic. 

### 2.5. Statistical Analysis

Data pertaining to embryonic development and mutation efficiency were evaluated using the analysis of variance (ANOVA) test followed by Fisher’s protected least significant difference (PLSD) test using STATVIEW (Abacus Concepts, Inc., Berkeley, CA, USA) [27]. All percentage data were subjected to arcsin transformation before ANOVA tests. The percentages of mutations within all blastocysts and samples of pooled embryos were analyzed using a chi-squared analysis with Yates’ correction. Differences with a probability value (*p*) of 0.05 or less were regarded as significant.

## 3. Results

**Figure 3:**
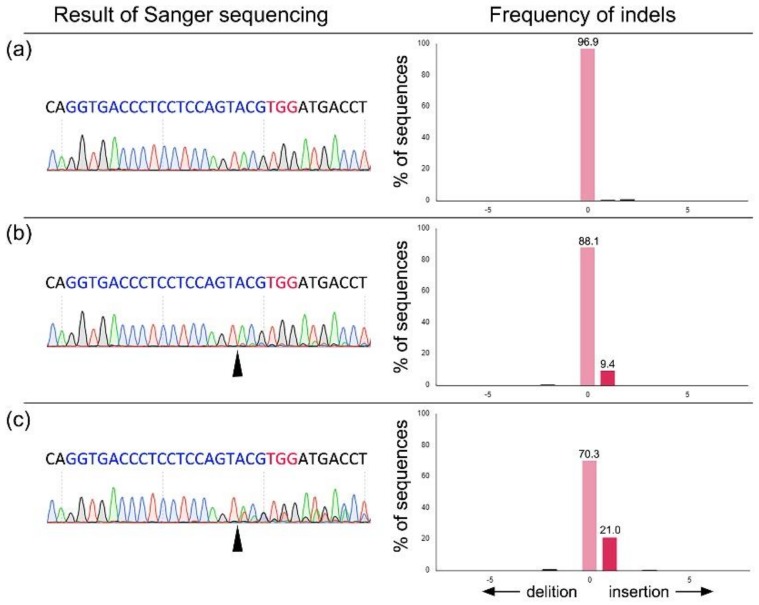
Representative result of Sanger sequencing analysis after the introduction of gRNA targeting the PERV *pol* gene and Cas9 protein, and the frequencies of indel mutation quantified by a Tracking of Indels by Decomposition (TIDE) analysis. Sanger sequencing results obtained from the blastocysts developed after the introduction of gRNA# 1 are shown. (**a**) Analysis of wild-type blastocyst. (**b**,**c**) Analysis of the genome-edited blastocyst. Example of the result showing relatively low (b; 9.4% of 1 bp insertion) or high (c; 21.0% of 1 bp insertion) indel frequency. Arrowhead indicates the Cas9 cleavage site. Nucleotides in blue color indicate target sequence of PERV#1 gRNA, and nucleotides in red color indicate protospacer adjacent motif (PAM) sequences.

We examined the effects of gRNA alone (Table 1) and in combination (Table 2) targeting the PERV *pol* gene on the development of embryos and their mutation. The electroporation treatment with gRNAs targeting the PERV *pol* gene decreased the rate of blastocyst formation in zygotes compared to untreated zygotes, irrespective of the type and combination of gRNA. In the embryos electroporated with each gRNA, the blastocyst formation rate in zygotes electroporated with gRNA3 was significantly lower (*p* < 0.05) than that in zygotes electroporated with gRNA1 (Table 1). Similarly, combining gRNA3 with the other gRNA significantly decreased the blastocyst formation rate as compared with the combination of gRNA1 and gRNA 2 (*p* < 0.05) (Table 2). When the mutation rates were assessed by sequencing the target sites of the PERV *pol* gene in individual blastocysts, the rates did not differ among the three groups, irrespective of the types or combinations of gRNA. Similarly, when the frequency of indel mutations (mutation efficiency) in mutant blastocysts was examined by TIDE (Figure 3), there were no significant between-group differences. 

Since we were unable to obtain a sufficient number of blastocysts from zygotes electroporated with gRNA3 (either alone or in combination), we examined the mutation rates and mutation efficiency of pooled embryos at the 2-to-8-cell stage (Table 3). The mutation rates in the samples of pooled embryos ranged from 80.0%–100% and were similar across all groups, irrespective of the types and combinations of gRNA. The mutation efficiency of mutant embryos in the single gRNA3 group was significantly higher (*p* < 0.05) than in the single gRNA2 group, but similar to that in the single gRNA1 group. However, the combination of gRNA3 with the other gRNA significantly increased the mutation efficiency as compared with the combination of gRNA1 and gRNA 2 (*p* < 0.05).

## 4. Discussion

The three major proteins encoded within the PERV genome are *gag, pol*, and *env*. The *pol* gene produces a reverse transcriptase that assists viral replication and infection [15]. Disruption of the PERV *pol* genes, which are necessary for viral replication in porcine kidney cells, using CRISPR/Cas9 could reduce the transmission of PERV to human cells by more than 1000-fold [15]. Targeting the *pol* gene did not interfere with normal placental function in pigs [28], suggesting that *pol* genes are ideal sites for gene editing. If the *pol* gene could be interrupted at a particular site, the virus would not be able to multiply inside the body of the pigs, improving the safety of xenotransplantation. We designed and tested three different constructs of gRNA that targeted the PERV *pol* gene to determine if these gRNAs could increase the mutation efficiency with suitable embryonic developmental competence.

We determined the relationship between embryonic development and the efficiency of target mutations in the PERV *pol* gene. The embryonic cleavage rate is a biological indicator of developmental potential, and the blastocyst formation rate of slow-cleaving embryos is low in mice [29]. Timing of first embryonic cleavage is also a positive indicator of the developmental potential of porcine in vitro fertilized zygotes [30]. In the present study, several cleaved embryos were monitored after electroporation with three gRNAs, either alone or in combination. Our results showed that the type of gRNA had no apparent effect on the embryonic cleavage rates, but affected the rate of blastocyst formation in zygotes. The blastocyst formation rate of zygotes electroporated with gRNA3 alone decreased compared to zygotes electroporated with gRNA1 alone. Moreover, the combination of gRNA3 with the other gRNA decreased the blastocyst formation rate of zygotes. These results are consistent with our previous report, which demonstrated that the type of gRNA affected the development of porcine embryos edited by the CRISPR/Cas9 system [17]. 

On the other hand, sequence features or type of gRNA could affect the success of the CRISPR/Cas9 targeting system [31]. Moreover, DNA cleavages by the CRISPR/Cas9 system at multiple PERV sites triggered DNA damage-induced senescence or apoptosis, affecting the growth of the mutated cells [16]. Previous studies reported that the number of copies of PERV genes differed relative to the breed, with commercial Western pigs containing more PERV copies than wild and native pigs [11,12,32,33]. The PCR titration analysis detected 32–64 *pol* copies in Landrace × Duroc crossbred pigs [34]. Therefore, when the CRISPR/Cas9 system edited porcine embryos from Western commercial breeds with gRNAs targeting the PERV *pol* gene, more DNA cleavages at multiple PERV sites seemed to occur. We used porcine zygotes derived from ovaries and semen in Western breeds, and subjected the zygotes to electroporation treatment with gRNAs targeting the PERV *pol* gene. The mutation efficiency in the mutant embryos at the 2-to-8-cell stage trended to be higher in the embryos electroporated with gRNA3 alone and in combination, which decreased the blastocyst formation rate of the zygotes. Since PERV exists in multiple copies in the pig genome, the high editing efficiency observed in the embryos electroporated with gRNA3 alone and in combination might induce the simultaneous cleavage of DNA, resulting in low embryonic development. Therefore, in Western breeds with more PERV copies, embryonic development may relate to the efficiency of target mutations in the PERV *pol* genes of the modified embryos. 

In conclusion, we found a negative relationship between embryonic development and the efficiency of target mutations in the PERV *pol* gene of porcine embryos. Further studies should attempt to elucidate additional technical points, including the type and combination of gRNAs targeting the *pol* gene. This information will enhance favorable outcomes related to PERV *pol* gene-edited pig production. 

## Figures and Tables

**Table 1:** Effects of gRNA targeting the PERV *pol* gene on the development of electroporated zygotes and the mutation in the blastocysts ^†^.

gRNA ^††^	No. of Oocytes Examined	No. (Mean ± SEM) of Embryos		No. of Blastocysts Examined	No. (Mean) of Mutated Blastocysts ^†††^	Total Mutation Efficiency (Mean ± SEM) ^††††^
		Cleaved	Developed to Blastocysts			
Control	184	159 (86.1± 1.5)	41 (21.5 ± 3.9) ^a^	-	-	-
gRNA1	267	227 (85.1 ± 2.6)	22 (9.2 ± 3.4) ^b^	18	13 (72.2)	12.4 ± 2.2
gRNA2	263	220 (83.9 ± 2.4)	11 (4.3 ± 1.6) ^b,c^	10	5 (50.0)	11.8 ± 2.1
gRNA3	263	224 (85.5 ± 3.1)	1 (0.4 ± 0.4) ^c^	1	1 (100)	18.1

^†^ Five replicate trials were completed. ^††^ Electroporation was performed by 1 ms and five pulses at 25 V/mm. As a control, the zygotes were cultured without performing electroporation. ^†††^ The proportions were calculated by dividing the number of mosaic mutant blastocysts by the total number of sequenced blastocysts. ^††††^ The mean proportions represent the proportion of indel mutation events in mosaic mutant blastocysts determined by the TIDE bioinformatics package. ^a–c^ Values with different superscripts in the same column are significantly different (*p* < 0.05).

**Table 2:** Effects of gRNAs in combination targeting the PERV *pol* gene on the development of electroporated zygotes and mutations in the blastocysts ^†^.

gRNA ^††^	No. of Oocytes Examined	No. (Mean ± SEM) of Embryos		No. of Blastocysts Examined	No. (Mean) of Mutated Blastocysts ^†††^	Total Mutation Efficiency (Mean ± SEM) ^††††^
		Cleaved	Developed to Blastocysts			
Control	157	124 (79.4 ± 3.2) ^a^	28 (14.4 ± 1.5) ^a^	-	-	-
gRNA1 & gRNA2	230	204 (88.9 ± 2.1) ^b^	11 (4.7 ± 1.0) ^b^	10	8 (80.0)	11.0 ± 3.1
gRNA1 & gRNA3	197	146 (74.0 ± 3.2) ^a^	2 (1.1 ± 0.6) ^c^	2	1 (50.0)	15.9
gRNA2 & gRNA3	222	174 (78.8 ± 4.6) ^a^	0 (0) ^c^	-	-	-

^†^ Four replicate trials were carried out. ^††^ Electroporation was performed by 1 ms and five pulses at 25 V/mm. As a control, the zygotes were cultured without performing electroporation. ^†††^ The proportions were calculated by dividing the number of mosaic mutant blastocysts by the total number of sequenced blastocysts. ^††††^ The mean proportions represent the proportion of indel mutation events in mosaic mutant blastocysts determined by the TIDE bioinformatics package. ^a–c^ Values with different superscripts in the same column are significantly different (*p* < 0.05).

**Table 3:** Mutation rate and efficiency in pooled embryos at the 2-to-8-cell stage derived from zygotes that had been electroporated with three different gRNAs targeting the PERV *pol* gene, either alone or in combination.

gRNA ^††^	No. ofPooled Embryo Sample	No. (Mean) of Mutated Embryo Sample ^†^	Total Mutation Efficiency (Mean ± SEM) ^††^
gRNA1	10	10 (100)	32.9 ± 3.1 ^a,b^
gRNA2	9	8 (88.9)	17.1 ± 2.9 ^a^
gRNA3	8	8 (100)	44.0 ± 5.1 ^b^
gRNA1 & gRNA2	15	12 (80.0)	21.1 ± 4.1 ^a^
gRNA1 & gRNA3	16	14 (87.5)	59.9 ± 6.2 ^c^
gRNA2 & gRNA3	10	10 (100)	42.2 ± 5.8 ^b^

^†^ The proportions were calculated by dividing the number of mutated embryo sample by the total number of sequenced embryo sample. ^††^ The mean proportions represent the proportion of indel mutation events in the mutated embryo sample determined by the TIDE bioinformatics package. ^a–c^ Values with different superscripts in the same column are significantly different (*p* < 0.05).
